# PyEEG: An Open Source Python Module for EEG/MEG Feature Extraction

**DOI:** 10.1155/2011/406391

**Published:** 2011-03-29

**Authors:** Forrest Sheng Bao, Xin Liu, Christina Zhang

**Affiliations:** ^1^Department of Computer Science, Department of Electrical Engineering, Texas Tech University, Lubbock TX 79409-3104, USA; ^2^ECHO Labs, Nanjing University of Posts and Telecommunications, Nanjing 210003, China; ^3^Department of Physiology, McGill University, Montreal, QC, Canada H3G 1Y6

## Abstract

Computer-aided diagnosis of neural diseases from EEG signals (or other physiological signals that can be treated as time series, e.g., MEG) is an emerging field that has gained much attention in past years. Extracting features is a key component in the analysis of EEG signals. In our previous works, we have implemented many EEG feature extraction functions in the Python programming language. As Python is gaining more ground in scientific computing, an open source Python module for extracting EEG features has the potential to save much time for computational neuroscientists. In this paper, we introduce PyEEG, an open source Python module for EEG feature extraction.

## 1. Introduction

Computer-aided diagnosis based on EEG has become possible in the last decade for several neurological diseases such as Alzheimer's disease [1, 2] and epilepsy [3, 4]. Implemented systems can be very useful in the early diagnosis of those diseases. For example, traditional epilepsy diagnosis may require trained physicians to visually screen lengthy EEG records whereas computer-aided systems can shorten this time-consuming procedure by detecting and picking out EEG segments of interest to physicians [5, 6]. On top of that, computers can extend our ability to analyze signals. Recently, researchers have developed systems [3, 4, 7, 8] that can hopefully use (any) random interictal (i.e., non-seizure) EEG records for epilepsy diagnosis in instances that are difficult for physicians to make diagnostic decisions with their naked eyes. In addition to analyzing existing signals, this computer-based approach can help us model the brain and predict future signals, for example, seizure prediction [9, 10].

All the above systems rely on characterizing the EEG signal into certain features, a step known as feature extraction. EEG features can come from different fields that study time series: power spectral density from signal processing, fractal dimensions from computational geometry, entropies from information theory, and so forth. An open source tool that can extract EEG features would benefit the computational neuroscience community since feature extraction is repeatedly invoked in the analysis of EEG signals. Because of Python's increasing popularity in scientific computing, and especially in computational neuroscience, a Python module for EEG feature extraction would be highly useful. In response, we have developed PyEEG, a Python module for EEG feature extraction, and have tested it in our previous epileptic EEG research [3, 8, 11].

Compared to other popular programming languages in scientific computing such as C++ or MATLAB, Python is an open source scripting language of simple syntax and various high-level libraries (for detailed advantages of Python, read http://www.python.org/about/), such as Scipy (http://www.scipy.org/) which allows users to run MATLAB codes after slight modification. There have been several popular open source Python projects in the neuroimaging community already, such as NIPY (http://nipy.sourceforge.net/). However, in neural physiology community, Python is not yet quite popular. As we are not aware of any open source tools in Python (or other programming languages) that can extract EEG features as mentioned above, we introduce and release PyEEG in this paper.

Though originally designed for EEG, PyEEG can also be used to analyze other physiological signals that can be treated as time series, especially MEG signals that represent the magnetic fields induced by currents of neural electrical activities.

The rest of the paper is organized as follows. In Section 2, we introduce the framework of PyEEG.  Section 3 gives the definitions to compute EEG features. A tutorial of applying PyEEG onto a public real EEG dataset is given in Section 4.  Section 5 concludes the paper.

## 2. Main Framework

**Figure 1:**
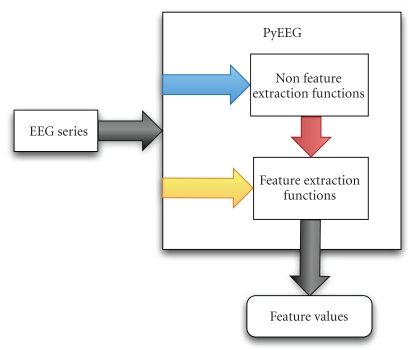
PyEEG framework.

PyEEG's target users are programmers (anyone who writes programs) working on computational neuroscience.  Figure 1 shows its framework. PyEEG is a Python module that focuses only on extracting features from EEG/MEG segments. Therefore, it does not contain functions to import data of various formats or export features to a classifier. This is due to the modularity and composition principles of building open source software which indicate that small programs that can work well together via simple interfaces are better than big monolithic programs. Since open source tools like EEG/MEG data importers (e.g., EEGLab, Biosig, etc.) and classifier front-ends are already available, there is no need for us to reinvent the wheel. Users can easily hook PyEEG up with various existing open source software to build toolchains for their EEG/MEG research.

PyEEG consists of two sets of functions.

1. *Preprocessing functions*, which do not return any feature values. Only two such functions have been implemented so far. embed_seq() builds embedding sequence (from given lag and embedding dimension) and first_order_diff() computes first-order differential sequence. One can build differential sequences of higher orders by repeatedly applying first-order differential computing.
2. *Feature extraction functions*, that return feature values. These are listed in Table 1.

PyEEG only uses functions in standard Python library and SciPy, the *de facto* Python module for scientific computing. PyEEG does not define any new data structure, but instead uses only standard Python and NumPy data structures. The reason is that we want to simplify the use of PyEEG, especially for users without much programming background. The inputs of all functions are a time sequence as a list of floating-point numbers and a set of optional feature extraction parameters. Parameters have default values. The output of a feature extraction function is a floating-point number if the feature is a scalar or a list of floating-point numbers (a vector) otherwise. Details about functions are available in the PyEEG reference guide at http://PyEEG.SourceForge.net/.

## 3. Supported Feature Extraction

In this section, we detail the definitions and computation procedures to extract EEG features (as shown in Table 1) in PyEEG. Since there are many parameters and various algorithms for one feature, the numerical value of a feature extracted by PyEEG may be different from that extracted by other toolboxes. Users may need to adjust our code or use non-default values for the parameters in order to meet their needs. Please note that the index of an array or a vector starts from 1 rather than 0 in this section.

### 3.1. Power Spectral Intensity and Relative Intensity Ratio

To a time series [*x*_1_, *x*_2_,…, *x*_*N*_], denote its Fast Fourier Transform (FFT) result as [*X*_1_, *X*_2_,…, *X*_*N*_]. A continuous frequency band from *f*_low_ to *f*_up_ is sliced into *K* bins, which can be of equal width or not. Boundaries of bins are specified by a vector *band* = [*f*_1_, *f*_2_,…, *f*_*K*_], such that the lower and upper frequencies of the *i*th bin are *f*_*i*_ and *f*_*i*+1_, respectively. Commonly used unequal bins are EEG/MEG rhythms, which are, *δ* (0.5–4 Hz), *θ* (4–7 Hz), *α* (8–12 Hz), *β* (12–30 Hz), and *γ* (30–100 Hz). For these bins, we have *band* = [0.5,4, 7,12,30,100].

The Power Spectral Intensity (PSI) [12] of the *k*th bin is evaluated as

(1) $${\text{PSI}}_{k}=\overset{\left\lfloor N(f_{k+1}/f_{\text{s}})\right\rfloor }{\underset{i=\left\lfloor N(f_{k}/f_{\text{s}})\right\rfloor }{\sum }}\left|X_{i}\right|,\;k=1,2,\ldots ,K-1,$$

where *f*_s_ is the sampling rate, and *N* is the series length.

Relative Intensity Ratio (RIR) [12] is defined on top of PSI

(2) $${\text{RIR}}_{j}=\frac{{\text{PSI}}_{j}}{\sum_{k=1}^{K-1}{\text{PSI}}_{k}},\;j=1,2,\ldots ,K-1.$$

PSI and RIR are both vector features.

### 3.2. Petrosian Fractal Dimension (PFD)

To a time series, PFD is defined as

(3) $$\begin{array}{c} \text{PFD}=\frac{{\log\;}_{10}N}{{\log\;}_{10}N+{\log\;}_{10}\left(N/\left(N+0.4N_{\delta }\right)\right)}, \end{array}$$

where *N* is the series length, and *N*_*δ*_ is the number of sign changes in the signal derivative [13]. PFD is a scalar feature.

### 3.3. Higuchi Fractal Dimension (HFD)

Higuchi's algorithm [14] constructs *k* new series from the original series [*x*_1_, *x*_2_,…, *x*_*N*_] by

(4) $$\begin{array}{c} x_{m},x_{m+k},x_{m+2k},\ldots ,x_{m+\lfloor \left(N-m\right)/k\rfloor k}, \end{array}$$

where *m* = 1,2,…, *k*.

For each time series constructed from (4), the length *L*(*m*, *k*) is computed by

(5) $$\begin{array}{c} L\left(m,k\right)=\frac{\sum_{i=2}^{\left\lfloor \left(N-m\right)/k\right\rfloor }\left|x_{m+ik}-x_{m+(i-1)k}\right|\left(N-1\right)}{\left\lfloor \left(N-m\right)/k\right\rfloor k}. \end{array}$$

The average length is computed as *L*(*k*) = [∑_*i*=1_^*k*^ *L*(*i*, *k*)]/*k*.

This procedure repeats *k*_max_ times for each *k* from 1 to *k*_max_, and then uses a least-square method to determine the slope of the line that best fits the curve of ln (*L*(*k*)) versus ln (1/*k*). The slope is the Higuchi Fractal Dimension. HFD is a scalar feature.

### 3.4. Hjorth Parameters

To a time series [*x*_1_, *x*_2_,…, *x*_*N*_], the Hjorth mobility and complexity [15] are, respectively, defined as $\sqrt{M2/\text{TP}}$ and $\sqrt{\left(M4\cdot \text{TP}\right)/\left(M2\cdot M2\right)}$, where TP = ∑*x*_*i*_/*N*, *M*2 = ∑*d*_*i*_/*N*, *M*4 = ∑(*d*_*i*_−*d*_*i*−1_)^2^/*N*, and *d*_*i*_ = *x*_*i*_ − *x*_*i*−1_. Hjorth mobility and complexity are both scalar features.

### 3.5. Spectral Entropy

The spectral entropy [16] is defined as follows

(6) $$\begin{array}{c} H=-\frac{1}{\log\;\;\left(K\right)}\overset{K}{\underset{i=1}{\sum }}{\text{RIR}}_{i}\log\;\;{\text{RIR}}_{i}, \end{array}$$

where RIR_*i*_ and *K* are defined in (2). Spectral entropy is a scalar feature.

### 3.6. SVD Entropy

Reference [17] defines an entropy measure using Singular Value Decomposition (SVD). Let the input signal be [*x*_1_, *x*_2_,…, *x*_*N*_]. We construct delay vectors as

(7) $$\begin{array}{c} y\left(i\right)=\left[x_{i},x_{i+\tau },\ldots ,x_{i+(d_{E}-1)\tau }\right], \end{array}$$

where *τ* is the delay and *d*_*E*_ is the embedding dimension. In this paper, *d*_*E*_ = 20 and *τ* = 2. The embedding space is then constructed by

(8) $$\begin{array}{c} Y={\left[y\left(1\right),y\left(2\right),\ldots ,y\left(N-\left(d_{E}-1\right)\tau \right)\right]}^{T}. \end{array}$$

The SVD is then performed on matrix *Y* to produce *M* singular values, *σ*_1_,…, *σ*_*M*_, known as the singular spectrum.

The SVD entropy is then defined as

(9) $$\begin{array}{c} H_{\text{SVD}}=-\overset{M}{\underset{i=1}{\sum }}{\bar{\sigma }}_{i}{\log\;}_{2}{\bar{\sigma }}_{i}, \end{array}$$

where *M* is the number of singular values and ${\bar{\sigma }}_{1},\ldots ,{\bar{\sigma }}_{M}$ are normalized singular values such that ${\bar{\sigma }}_{i}=\sigma_{i}/\sum_{j=1}^{M}\sigma_{j}$. SVD entropy is a scalar feature.

### 3.7. Fisher Information

The Fisher information [18] can be defined in normalized singular spectrum used in (9)

(10) $$\begin{array}{c} I=\overset{M-1}{\underset{i=1}{\sum }}\frac{{\left({\bar{\sigma }}_{i+1}-{\bar{\sigma }}_{i}\right)}^{2}}{{\bar{\sigma }}_{i}}. \end{array}$$

Fisher information is a scalar feature.

### 3.8. Approximate Entropy

Approximate entropy (ApEn) is a statistical parameter to quantify the regularity of a time series [19].

ApEn is computed by the following steps.

Let the input signal be [*x*_1_, *x*_2_,…, *x*_*N*_].
Build subsequence *x*(*i*, *m*) = [*x*_*i*_, *x*_*i*+1_,…, *x*_*i*+*m*−1_] for 1 ≤ *i* ≤ *N* − *m*, where *m* is the length of the subsequence. In [7], *m* = 1,2, or 3.
Let *r* represent the noise filter level, defined as *r* = *k* × SD for *k* = 0,0.1,0.2,…, 0.9.
Build a set of subsequences {*x*(*j*, *m*)} = {*x*(*j*, *m*) | *j* ∈ [1..*N* − *m*]}, where *x*(*j*, *m*) is defined in step 2.
For each *x*(*i*, *m*)∈{*x*(*j*, *m*)}, compute
  (11) $$\begin{array}{c} C\left(i,m\right)=\frac{\sum_{j=1}^{N-m}k_{j}}{N-m}, \end{array}$$
  where
  (12) $$k_{j}=\{\begin{array}{cc} 1 & \text{if}\;\;\left|x\left(i,m\right)-x\left(j,m\right)\right|<r,\\ 0 & \text{otherwise}. \end{array}$$
  (13) $$\text{ApEn}\left(m,r,N\right)=\frac{1}{N-M}\left[\overset{N-m}{\underset{i=1}{\sum }}\ln\;\frac{C\left(i,m\right)}{C\left(i,m+1\right)}\right].$$

ApEn is a scalar feature.

### 3.9. Detrended Fluctuation Analysis

Detrended Fluctuation Analysis (DFA) is proposed in [20].

The procedures to compute DFA of a time series [*x*_1_, *x*_2_,…, *x*_*N*_] are as follows.

1. First integrate *x* into a new series *y* = [*y*(1),…, *y*(*N*)], where $y(k)=\sum_{i=1}^{k}\left(x_{i}-\bar{x}\right)$ and $\bar{x}$ is the average of *x*_1_, *x*_2_,…, *x*_*N*_.
2. The integrated series is then sliced into boxes of equal length *n*. In each box of length *n*, a least-squares line is fit to the data, representing the *trend* in that box. The *y* coordinate of the straight line segments is denoted by *y*_*n*_(*k*).
3. The root-mean-square fluctuation of the integrated series is calculated by $F(n)=\sqrt{(1/N)\sum_{k=1}^{N}{\left[y\left(k\right)-y_{n}\left(k\right)\right]}^{2}}$, where the part *y*(*k*) − *y*_*n*_(*k*) is called detrending.
4. The fluctuation can be defined as the slope of the line relating log  *F*(*n*) to log  *n*.

DFA is a scalar feature.

### 3.10. Hurst Exponent

The hurst exponent (HURST) [21] is also called Rescaled Range statistics (R/S). To calculate the hurst exponent for time series *X* = [*x*_1_, *x*_2_,…, *x*_*N*_], the first step is to calculate the accumulated deviation from the mean of time series within range *T*

(14) $$\begin{array}{c} X\left(t,T\right)=\overset{t}{\underset{i=1}{\sum }}\left(x_{i}-\bar{x}\right),\;\text{where}\;\bar{x}=\frac{1}{T}\overset{T}{\underset{i=1}{\sum }}x_{i},\;\;t\in \left[1..N\right]. \end{array}$$

Then, R(*T*)/S(*T*) is calculated as

(15) $$\begin{array}{c} \frac{\text{R}\left(T\right)}{\text{S}\left(T\right)}=\frac{\max\;\;\left(X\left(t,T\right)\right)-\min\;\;\left(X\left(t,T\right)\right)}{\sqrt{\left(1/T\right)\sum_{t=1}^{T}{\left[x\left(t\right)-\bar{x}\right]}^{2}}}. \end{array}$$

The Hurst Exponent is obtained by calculating the slope of the line produced by ln  (R(*n*)/S(*n*)) versus ln  (*n*) for *n* ∈ [2..*N*]. Hurst Exponent is a scalar feature.

## 4. Using PyEEG on Real Data

In this section, we use PyEEG on a real EEG dataset to demonstrate its use in everyday research.

The dataset (http://epileptologie-bonn.de/cms/front_content.php?idcat=193&lang=3), from Klinik für Epileptologie, Universität Bonn, Germany [22], has been widely used in previous epilepsy research. In total, there are five sets, each containing 100 single-channel EEG segments. Each segment has 4096 samples. Data in sets A and B are extracranial EEGs from 5 healthy volunteers with eyes open and eyes closed, respectively. Sets C and D are intracranial data over interictal periods while Set E over ictal periods. Segments in D are from within the epileptogenic zone, while those in C are from the hippocampal formation of the opposite hemisphere of the brain. Sets C, D, and E are composed from EEGs of 5 patients. The data had a spectral bandwidth of 0.5–85 Hz. Please refer to [22] for more details.

Using PyEEG is like using any other Python module. Users simply need to import PyEEG and then call its functions as needed. PyEEG is provided as a single Python file. Therefore, it only needs to be downloaded and placed under a directory on Python module search paths, such as the working directory. Alternatively, PYTHONPATH environment variable can be set to point to the location of PyEEG.

On Python interpreter, we first import PyEEG and load the data

>>> import pyeeg
>>> fid = open('Z001.txt', 'r')
>>> tmp = fid.readlines()
>>> data = [float(k) for k in tmp]

where Z001.txt is the first segment in set A. The data type of data is list. After loading EEG data, we can use PyEEG to extract features as follows (using all default parameters):

>>> DFA = pyeeg.dfa(data)
>>> DFA
0.81450526948129354
>>> Hurst_Exponent = pyeeg.hurst(data)
>>> Hurst_Exponent
0.68053321812240675
>>> PFD = pyeeg.pfd(data)
>>> PFD
0.58651018327048932

**Figure 2:**
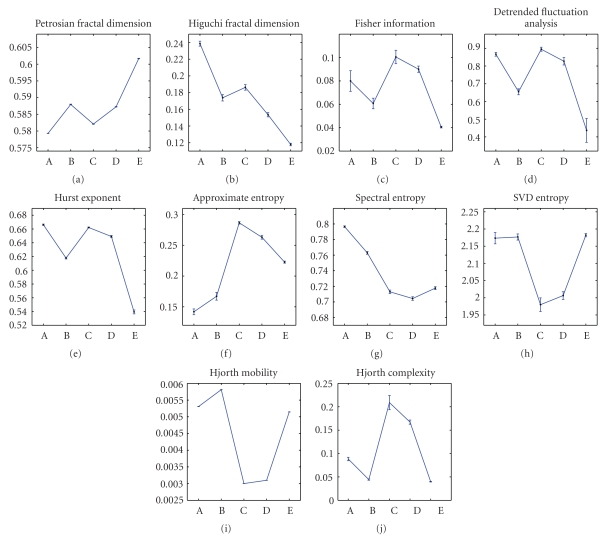
Distributions of ten features extracted by PyEEG in each set.

**Figure 3:**
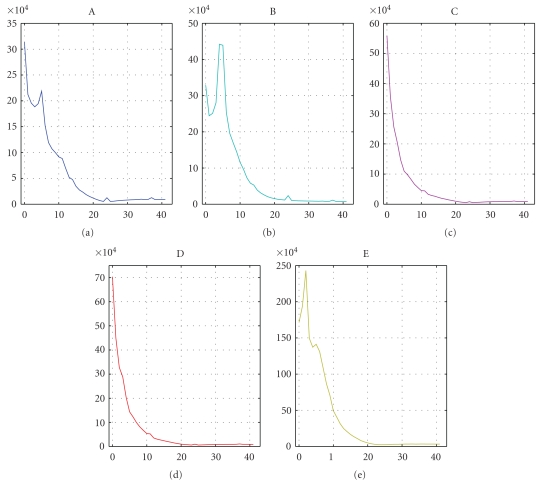
Average PSI of each set. Note that the scale in *y*-axis of set E is much larger than that of other sets.

Due to space limitations, we are not able to print all feature values of all EEG segments. Instead, we visualize the averages of the features (except RIR and PSI) within each of the five sets in Figure 2. Error bars represent the variances of features in each set. PSIs for five sets are plotted in Figure 3. Users can replot these pictures and get averages of features on Python interpreter by a testing script (http://code.google.com/p/pyeeg/wiki/TestScript) from our project website.

From Figures 2 and 3, we can see that healthy, interictal, and ictal EEG signals have different distributions for most features.  Table 2 lists parameters used in this experiment.

## 5. Discussion and Future Development

So far, we have listed features that can be extracted by PyEEG and their definitions. Our implementation sticks on their definitions precisely even though faster algorithms may exist. There are many other EEG features, such as Lyapunov Exponents, that have not been yet implemented in PyEEG. More EEG features will be added into PyEEG in the future while we finish unit testing and documentation for each function. In personal emails, some open source projects, such as ConnectomeViewer (http://www.connectomeviewer.org/viewer) and NIPY/PBrain (http://nipy.sourceforge.net/pbrain/), have expressed the interest in including PyEEG into their code. Therefore, we will keep maintaining PyEEG as long as it can benefit the entire computational neuroscience community.

##  Availability

 The software is released under GNU GPL v.3 at Google Code: http://code.google.com/p/pyeeg/. No commercial software is required to run PyEEG. Because Python is cross-platform, PyEEG can run on Linux, Mac OS, and Windows.

## Figures and Tables

**Table 1:** PyEEG-supported features and extraction functions with their return types.

Feature name	Function name	Return type
Power Spectral Intensity (PSI) and Relative Intensity Ratio (RIR)	bin_power()	Two 1-D vectors
Petrosian Fractal Dimension (PFD)	pdf()	A scalar
Higuchi Fractal Dimension (HFD)	hfd()	A scalar
Hjorth mobility and complexity	hjorth()	Two scalars
Spectral Entropy (Shannon's entropy of RIRs)	spectral_entropy()	A scalar
SVD Entropy	svd_entropy()	A scalar
Fisher Information	fisher_info()	A scalar
Approximate Entropy (ApEn)	ap_entropy()	A scalar
Detrended Fluctuation Analysis (DFA)	dfa()	A scalar
Hurst Exponent (Hurst)	hurst()	A scalar

**Table 2:** Values of parameters used in our example.

Parameter name	Value	In feature(s)
*k*_max _	5	HFD

*τ*	4	SVD Entropy
*d*_*E*_	10	Fisher Information

*r*	0.3*σ* ^1^	ApEn
*m*	10	

*f*_*s*_	173	Spectral Entropy
*ba* *nd*	[1,3, 5,…, 85]	PSI and RIR

^1^ *σ* is the standard deviation of the EEG segment.
